# SADASNet: A Selective and Adaptive Deep Architecture Search Network with Hyperparameter Optimization for Robust Skin Cancer Classification

**DOI:** 10.3390/diagnostics15050541

**Published:** 2025-02-24

**Authors:** Günay İlker, İnik Özkan

**Affiliations:** Department of Computer Engineering, Tokat Gaziosmanpaşa University, Tokat 60250, Turkey; ozkan.inik@gop.edu.tr

**Keywords:** HAM10000, CNN, PSO, hyperparameter, optimization, GAN, skin cancer

## Abstract

**Background/Objectives:** Skin cancer is a major public health concern, where early diagnosis and effective treatment are essential for prevention. To enhance diagnostic accuracy, researchers have increasingly utilized computer vision systems, with deep learning-based approaches becoming the primary focus in recent studies. Nevertheless, there is a notable research gap in the effective optimization of hyperparameters to design optimal deep learning architectures, given the need for high accuracy and lower computational complexity. **Methods:** This paper puts forth a robust metaheuristic optimization-based approach to develop novel deep learning architectures for multi-class skin cancer classification. This method, designated as the SADASNet (Selective and Adaptive Deep Architecture Search Network by Hyperparameter Optimization) algorithm, is developed based on the Particle Swarm Optimization (PSO) technique. The SADASNet method is adapted to the HAM10000 dataset. Innovative data augmentation techniques are applied to overcome class imbalance issues and enhance the performance of the model. The SADASNet method has been developed to accommodate a range of image sizes, and six different original deep learning models have been produced as a result. **Results:** The models achieved the following highest performance metrics: 99.31% accuracy, 97.58% F1 score, 97.57% recall, 97.64% precision, and 99.59% specificity. Compared to the most advanced competitors reported in the literature, the proposed method demonstrates superior performance in terms of accuracy and computational complexity. Furthermore, it maintains a broad solution space during parameter optimization. **Conclusions:** With these outcomes, this method aims to enhance the classification of skin cancer and contribute to the advancement of deep learning.

## 1. Introduction

Skin cancer is a major global health concern, classified into three primary types: melanoma, basal cell carcinoma, and squamous cell carcinoma. Among these, melanoma is the most aggressive and lethal, often identified through changes in moles. Early detection is critical for successful treatment, yet visual examination alone remains insufficient and prone to misdiagnosis [1]. Dermatologists traditionally use the ABCD rule, which evaluates Asymmetry, Border irregularity, Color variation, and Diameter, to assess suspicious pigmented lesions [2]. While this rule provides a structured diagnostic approach, it relies on subjective interpretation and may not always be effective, particularly in early-stage melanoma or atypical cases. Challenges such as inexperience, complex lesion patterns, and inconsistent training further complicate the diagnostic process. To overcome these limitations, automated diagnostic systems leveraging computer vision and machine learning algorithms have gained significant attention [3]. CNN-based architectures offer a more objective and reliable approach, surpassing traditional methods in classification accuracy and robustness. Recent studies indicate that CNNs remain the dominant approach, yet machine learning, expert systems, and multimodal models are increasingly being integrated to enhance classification performance [4,5]. Research on diverse datasets has highlighted the importance of data quality and standardization, demonstrating their direct impact on model reliability. Beyond deep learning models, optimization techniques are now being combined with CNN architectural search to further enhance performance. Evolutionary methodologies, hyperparameter optimization, and model search strategies are employed to develop more efficient and high-performing CNN architectures [6,7,8,9]. These techniques refine feature selection and classification, ultimately improving overall model accuracy and enabling automated architecture discovery [10]. Despite advancements, ethical concerns, dataset biases, and generalization issues remain key barriers to the clinical adoption of AI-based dermatological systems, with data quality and the lack of regulatory frameworks affecting reliability [11].

### 1.1. Related Work

A review of the literature reveals that recent studies have predominantly focused on Skin Cancer and that Hyperparameter Optimization (HPO) methods have started to be used as a powerful tool to support research in this field, as in many other domains. Each section highlights significant studies emphasizing advancements in skin cancer detection and classification.

Skin Cancer: Skin cancer detection and classification have become critical areas of research, with recent advancements in deep learning and artificial intelligence offering innovative solutions for improved accuracy and reliability. Reference [12] proposed a two-stage GAN-based method called “Self-Transfer GAN (STGAN)” to improve skin cancer diagnosis. STGAN learns global information from each class and combines this information with class-based features to produce high-quality skin lesion images to solve data imbalance problems. The model was tested on the HAM10000 [13] dataset and achieved an accuracy of 98.23%, overcoming issues such as mode collapse due to class scarcity. Moreover, the STGAN-based framework also achieved impressive results in performance metrics such as precision and accuracy. Reference [14] proposed a mask-guided deep learning approach for skin lesion classification, integrating CNNs and Vision Transformers (ViTs) with Grad-CAM and Grad-CAM++ for interpretability. Optimization techniques such as data augmentation and hyperparameter tuning improved model performance. Trained on the HAM10000 dataset, the model achieved 98.32% accuracy, 95.92% sensitivity, and 99.01% specificity. Reference [15] proposed an explainable artificial intelligence (XAI)-based computer-aided diagnosis system called “Skin-CAD” to improve the reliability of skin cancer (SC) diagnosis. Skin-CAD classifies skin lesions into two main classes, benign and malignant, and seven subclasses using four different CNN topologies. The system provides a more efficient training process by extracting features from two different deep layers and reducing their size with PCA. In addition, visual annotations with the LIME method help dermatologists to better understand the model predictions. Skin-CAD, Skin Cancer: Malignant vs. Benign dataset with 97.2% accuracy and HAM10000 dataset with 96.5% accuracy. Reference [16] proposes a Wavelet Transform-based Deep Residual Neural Network (WT-DRNNet) model for skin cancer diagnosis. This model aims to classify skin lesions more accurately using techniques such as wavelet transformation, pooling, and normalization. The experiments were conducted on ISIC2017 and HAM10000 datasets and the proposed model achieved successful results with high accuracy rates ranging from 96.91% to 95.73%. Reference [17] proposed a dual optimization-based deep learning network called “DODL net” to support early detection of skin cancer. The DODL net uses U-net to separate specific regions of images and includes dual optimization algorithms such as Bacterial Foraging Optimization (BFO) and Particle Swarm Optimization (PSO) for feature extraction. The performance of this model was evaluated for the HAM10000 dataset with a high accuracy rate of 98.76%.

Hyperparameter Optimization (HPO): Machine learning and deep learning models contain various hyperparameters that need to be optimized to achieve the best results. Therefore, hyperparameter optimization is of great importance, and various techniques have been developed in this field. Reference [18] optimized the SqueezeNet architecture using the Bald Eagle Search (BES) algorithm for skin cancer diagnosis. This model achieved impressive results, including high accuracy (98.37%), specificity (96.47%), and sensitivity (100%). I-HGS-ResNet50 [12,19] was developed by optimizing the ResNet50 model using the Hunger Games Search (I-HGS) algorithm for brain tumor classification. It achieved approximately 99.8% accuracy across three different datasets, surpassing other deep learning models. Reference [20] proposed a Simplified Swarm Optimization (SSO) algorithm for hyperparameter optimization. Experiments conducted on the LeNet model demonstrated that this method outperformed previous models and showed its applicability to more complex architectures. Reference [21] reduced the trial-and-error process in hyperparameter optimization using the Taguchi method to identify key hyperparameters. Analysis on a breast cancer dataset achieved an accuracy of 83.19%. HPO provides a broad framework that also encompasses Neural Architecture Search (NAS) techniques. Reference [9] introduced Efficient Neural Architecture Search (ENAS), an efficient architecture search method. ENAS achieved effective results while consuming significantly fewer GPU hours and required no additional post-processing. Reference [6] developed a Particle Swarm Optimization (PSO)-based CNN architecture search method for environmental sound classification. Architectures optimized with the PSO algorithm achieved superior results compared to previous studies. Reference [22] presented the EST-NAS method to address local minima and performance issues in NAS. This method combines an evolutionary strategy with gradient-based search, achieving competitive performance across various datasets and search spaces.

### 1.2. Motivation

In the literature, different state-of-the-art architectures have been used for skin cancer diagnosis. However, the development of new architectures based on optimization, together with performance metrics from both state-of-the-art and emerging CNN models, indicates a need for the development of new architectural models to enhance the accuracy of multi-class skin cancer diagnosis. For this purpose, it is considered to obtain CNN models with serial and parallel layer structures with high accuracy and low computational cost at the same time. To this end, the main motivation of this study is to develop a new approach to NAS with hyperparameter optimization. This approach aims to automatically generate CNN models with specially designed serial and parallel layer structures. Additionally, the optimal image size for the input layer of the CNN is investigated using the dataset employed for skin cancer classification. This study aims to contribute to the efforts of dermatologists and healthcare professionals in skin cancer classification, as well as to the advancement of deep learning in architecture search and hyperparameter optimization.

### 1.3. Contribution

- Automated CNN Architecture Design and Hyperparameter Optimization: The SADASNet algorithm introduces an automated approach to designing Convolutional Neural Network (CNN) architectures, eliminating the need for manual design processes. By integrating Particle Swarm Optimization (PSO), SADASNet simultaneously optimizes both the network architecture and layer hyperparameters, ensuring high sensitivity and performance in skin cancer classification tasks. This approach significantly reduces the time and expertise required to develop effective models.
- Robust Neural Architecture Search (NAS) Framework: SADASNet leverages PSO’s rapid convergence and model diversity capabilities to create a robust NAS framework. Unlike traditional methods, SADASNet directly evaluates the true performance of candidate architectures through independent training principles, addressing common evaluation challenges. This results in more accurate and reliable models for skin cancer classification.
- High Accuracy and Early Detection Support: The proposed method achieves state-of-the-art performance, with an accuracy of 99.31% on the HAM10000 dataset. This high level of accuracy supports more reliable skin cancer classification, which is critical for early detection and effective treatment.
- Class Imbalance Mitigation Using GANs: To address the challenge of class imbalance in skin cancer datasets, SADASNet employs Generative Adversarial Networks (GANs) for synthetic data generation. This approach enhances dataset diversity and balance, leading to improved model generalization and performance.
- Accessibility for Non-Experts: SADASNet provides an automated and user-friendly solution for researchers and healthcare professionals with limited expertise in deep learning. By simplifying the model development process, it enables broader participation in advancing skin cancer diagnosis and classification.

## 2. Methodology

### 2.1. Preliminary Preparation

In this section, the following methods are briefly discussed: Deep learning, CNN, GAN, PSO, model performance metrics, dataset, and balancing technique.

#### 2.1.1. Deep Learning

Deep learning (DL) is a machine learning method utilized for tasks such as classification, recognition, and prediction. The method of deep learning involves the use of a multilayer artificial neural network to solve a wide variety of problems. These networks are designed to process data of different types, including audio, video, and other forms of data, using multilayer architectures. The design of deep learning architectures can vary depending on the characteristics of the data, including its type, size, volume, structure, and parameters. Therefore, the choice of algorithm is critical. There are several types of deep learning approaches, including CNN and Recurrent Neural Networks (RNNs) [23].

##### CNN

**Figure 1:**
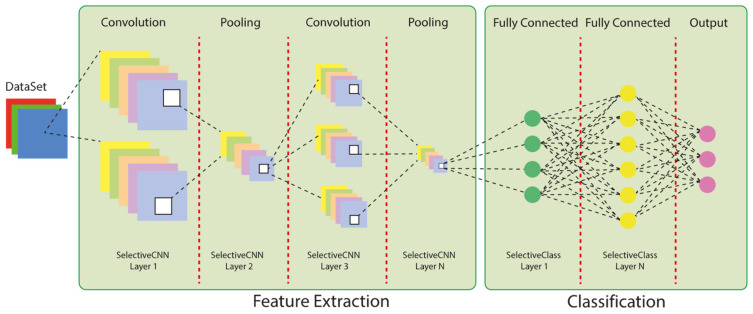
Steps for skin cancer classification using CNN.

CNN represents a significant advancement in image processing technology, exhibiting superior capabilities compared to traditional neural networks. CNNs are particularly well-suited for digital imaging applications due to their weight-sharing and sparse connectivity properties among image pixels. These networks can be enhanced through the incorporation of back-propagation, learning algorithms, and regularization methods. The fundamental layers of a CNN include convolution, pooling, and fully connected layers. The convolution layer is responsible for organizing a set of task-specific weights, while the pooling layers provide a means of reducing the dimensions of the data. The results of these operations are then passed to the fully connected layers, where they are used for feature synthesis and classification. Figure 1 illustrates the basic CNN architecture, which is based on the convolution layer and contains weight parameters that are optimized for different applications [24].

##### GAN

GANs are defined as a DL framework based on the interaction between two artificial neural networks. In this framework, two networks are trained to generate new data by competing against each other. The initial network, termed the Generator, is responsible for producing novel data that closely resembles real data. The second network, known as the Discriminator, aims to discern the distinction between real and generated data. In this context, the Generator attempts to deceive the Discriminator by integrating the fake data it generates with the genuine data, whereas the Discriminator strives to discern the distinction between the two. GANs are based on two networks that improve their capabilities by competing with each other. GANs offer a wide range of applications, including the generation of realistic visual, audio, video, and text data. Additionally, GANs can be utilized to reduce the size of datasets, generate new types of data, and enhance the quality of existing data [25].

#### 2.1.2. PSO

PSO is an iterative optimization algorithm developed by [26]. The objective of this algorithm is to emulate the natural movements observed in flocks of birds and fish. Such animals are able to reach their goals with greater efficiency by moving in flocks. The PSO algorithm is composed of particles that collectively constitute a swarm. Each particle represents a potential solution to the problem at hand. Each particle carries three attributes: its position, velocity, and a fitness value determined by an optimization function. The velocity determines the direction of movement and distance of the particle, while the fitness value measures the quality of the particle. Particles update their position by communicating with other particles in the swarm. Each particle tracks its personal best position as well as the global best position within the swarm. The personal best represents the best solution that the particle can achieve individually, while the global best represents the best solution that all particles can collectively find.

The PSO is mathematically modeled for an optimization problem as follows:

(1) $$X_{i}\left(t+1\right)=X_{i}\left(t\right)+V_{i}\left(t+1\right)$$

(2) $$V_{i}\left(t+1\right)=\omega V_{i}\left(t\right)+c_{1}r_{1}(P_{i}\left(t\right)-X_{i}\left(t\right))+c_{2}r_{2}(G(t)-X_{i}\left(t\right))$$

In this context, $X_{i}\left(t\right)$ and $V_{i}\left(t\right)$ represent the position and velocity of the particle, respectively. $P_{i}\left(t\right)$ denotes the personal best position, G(t) signifies the global best position, ω is the weight factor, and $c_{1}$ and $c_{2}$ are random constants.

#### 2.1.3. Dataset

In this study, dermoscopic images were obtained from the HAM10000 dataset, published by the International Skin Image Collaboration (ISIC) [27]. Originally provided by Kaggle, the HAM10000 dataset comprises dermoscopic images of pigmented lesions, commonly referred to as skin cancer. These images were collected from diverse populations, resulting in a total of 10,015 images used for our experiments. After addressing data imbalance, the dataset was divided into training and testing sets. Specifically, 80% of the dataset was allocated for training, while the remaining 20% was reserved for testing. Furthermore, within the training set, 90% of the data were used for model training, and 10% was set aside for validation.

##### Balancing Dataset

**Figure 2:**
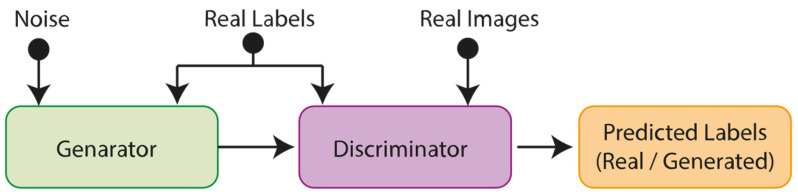
General structure of CGAN.

An initial examination of the HAM10000 dataset reveals a significant class imbalance, with 6705 images of Melanocytic nevi (nv), 1113 images of melanoma (mel), 1099 images of benign keratosis lesions (bkl), 514 images of basal cell carcinoma (bcc), 327 images of actinic keratoses (akiec), 142 images of vascular lesions (vasc), and 115 images of dermatofibroma (df). This inherent imbalance poses a risk of misclassification during model training. To address this limitation, GAN-based data augmentation was employed, specifically utilizing a Conditional GAN (CGAN) to oversample the minority classes. Unlike conventional GANs, CGANs generate new data instances conditioned on labeled inputs, thereby enabling the targeted augmentation of classes with fewer samples. As a result, the number of images for all classes except nv was increased to 6705, aligning them with the majority class. Figure 2 illustrates the general structure of the employed CGAN [28].

**Figure 3:**
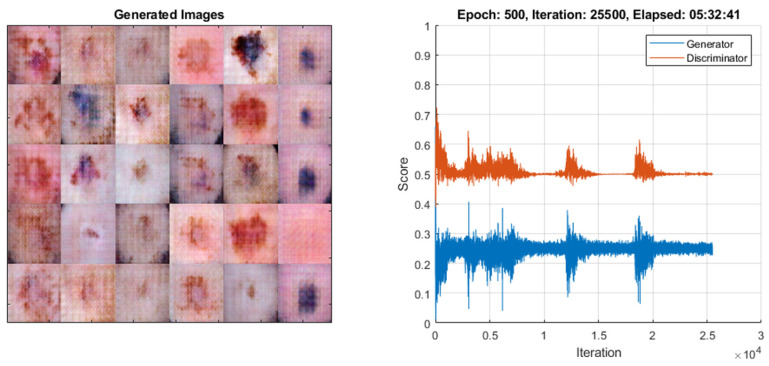
Training performance of the CGAN model.

The realism and validity of the data produced by GANs are critical, especially in the field of medical imaging. In this study, the scores obtained (Generator: 0.2542, Discriminator: 0.4976) reflect the balance between the diversity and realism of the images produced by the model. To minimize the risk of data leakage, the training and validation datasets were rigorously separated, and the model’s overdependence on the original dataset was checked with independent test cases. These methods increase the reliability of the model and contribute to the classification performance of the generated images. After training, the network was utilized to balance the dataset by increasing the data counts of underrepresented classes to match the count of the class with the highest data representation. Figure 3 illustrates the training performance of the CGAN model, highlighting the effectiveness of the approach in addressing dataset imbalance while maintaining image diversity and realism.

#### 2.1.4. Performance Metrics

**Figure 4:**
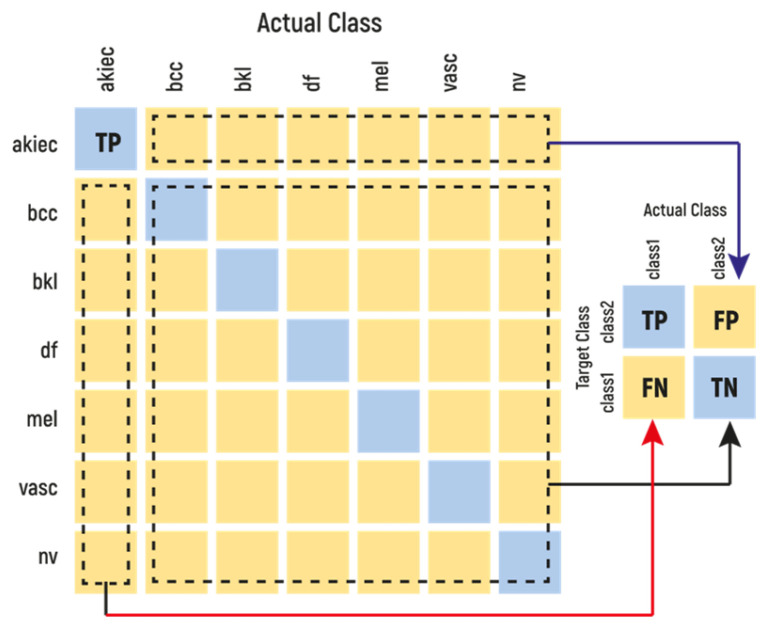
Confusion matrix.

In the field of machine learning, a confusion matrix (also known as an error matrix) is a tabular representation that provides a visual representation of the performance of an algorithm in statistical classification problems (see Figure 4). Each row of the matrix corresponds to instances in a true class, while each column corresponds to instances in a predicted class, facilitating the identification of instances where two classes are mixed [29].

The term accuracy is typically employed to quantify the performance of a model across all classes. This is particularly advantageous when all classes are of equal importance. Accuracy is calculated as the ratio of the number of correct predictions to the total number of predictions made.

(3) $$Precision=\frac{TP}{TP+FP}$$

(4) $$Specificity=\frac{TN}{TN+FP}$$

(5) $$Recall=\frac{TP}{TP+FN}$$

(6) $$F1_{Score}=\frac{2*Precision*Recall}{\left(Precision+Recall\right)}=\frac{2TP}{2TP+FP+FN}$$

(7) $$Accuracy=\frac{TP+TN}{TP+TN+FP+FN}$$

## 3. Proposed Method

**Figure 5:**
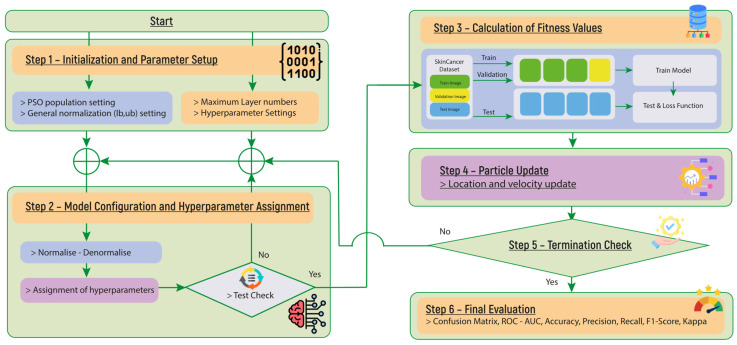
Proposed PSO-based SADASNet automatic model optimizer.

The proposed SADASNet framework, as illustrated in Figure 5, consists of six structured steps to efficiently optimize deep learning architectures. Step 1—Initialization and Parameter Setup: The process begins with defining the PSO population size, setting general normalization ranges [lb, ub], and specifying maximum layer numbers along with hyperparameter settings to establish the initial search space. Step 2—Model Configuration and Hyperparameter Assignment: In this stage, hyperparameters are assigned to each model candidate. The values are first normalized and later denormalized for proper model interpretation. A preliminary test check is conducted to determine hardware compatibility, ensuring that only valid models proceed to training. Step 3—Calculation of Fitness Values: The surviving models are trained on the skin cancer dataset, undergoing a train-validation-test split to evaluate their performance. During this process, the model’s loss function is computed, which serves as a key metric for optimization. Step 4—Particle Update: At this stage, the PSO algorithm updates the particle positions by adjusting their location and velocity based on the computed loss (fitness) values. Step 5—Termination Check: The optimization loop continues iteratively until a termination condition is met. This can be triggered by reaching the maximum number of iterations or by identifying a model that meets the predefined performance threshold. Step 6—Final Evaluation: Once the optimal model is identified, it is assessed using standard performance metrics, including the confusion matrix, ROC-AUC, accuracy, precision, recall, F1-score, and Kappa statistics, to ensure robustness and generalization.

### 3.1. Stages of the Proposed Method

Step 1. Initialization and Parameter Setup: The SADASNet algorithm begins by initializing the PSO search process and defining the hyperparameter search space.

**Figure 6:**
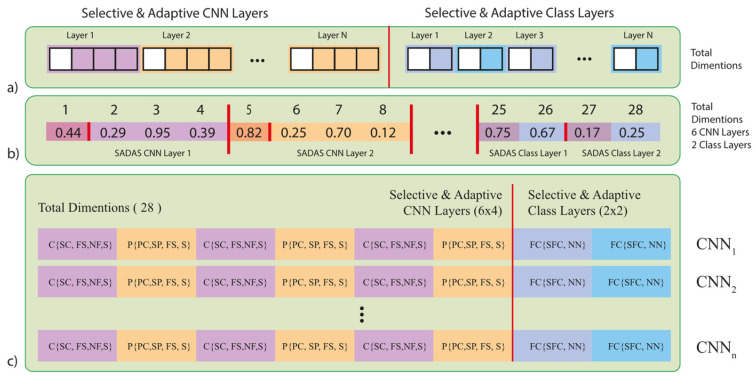
Selective and Adaptive Deep Architecture Search (SADASNet) Framework—(**a**) Structural representation, (**b**) Layer-wise parameter values, (**c**) Model variations.

1. PSO Initialization: The PSO algorithm is initialized with a user-defined swarm size and dimensionality based on the number of hyperparameters being optimized. Each particle’s initial position and velocity are randomly assigned within the normalized range [0, 1] (Figure 6a,b).

2. Hyperparameter Search Space: The maximum number of the layers for the SADASNet architecture is user-specified, constraining the search space. Hyperparameter ranges for each layer type are also defined as follows:

- Convolutional Layers: Standard convolutions use Convolution Control (CC) ∈ [1, 2] (presumably indicating presence or type), Filter Size (FS) ∈ [2, 8], Number of Filters (NF) ∈ [8, 512], and Stride (S) ∈ [1, 3]. Multi-branch convolutions utilize Number of Branches (NB) ∈ [2, 4], FS ∈ [2, 8], and NF ∈ [4, 128].
- Pooling Layers: Defined by Pooling Control (PC) ∈ [0, 1] (presence), Selective Pooling (SP) ∈ [1, 2] (type), Filter Size (FS) ∈ [1, 7], and Stride (S) ∈ [1, 3].
- Fully Connected Layers: Controlled by Fully Connected Control (FCC) ∈ [0, 1] (presence) and Number of Neurons (NN) ∈ [10, 512].

All hyperparameter ranges are user-definable, allowing for flexible adjustment of the search space complexity. The PSO algorithm normalizes all values to [0, 1] and maps them to actual hyperparameter values based on the SADASNet selection criteria (Algorithm 1).
**Algorithm 1** Pseudocode of SADASNet AlgortihmInput: ‘InputData’, ‘SCNN_Params’, ‘SClass_Params’
Output: ‘FinalOutput’
1. Initialize ‘InputData’, ‘SCNN’, and ‘SClass’ layers.
2. If ‘layerType == “single”’:  ‘ConvLayer’ = ‘Denormalize(params)’
 Else if ‘layerType == “double”’:
  ‘PoolLayer’ = ‘Denormalize(params)’
3. Process ‘SClass’ layer:
  ‘FCLayer’ = ‘Denormalize(params)’
4. Define ‘ConvLayer_Selection’:
 If ‘[1,1]’ is selected:
  ‘ConvLayer’ = ‘Denormalize(params, SC_Range, FS_Range, NF_Range, S_Range)’
   ‘SC ∈ [1,2]’, ‘FS ∈ [2,8]’, ‘NF ∈ [8,512]’, ‘S ∈ [1,3]’
 Else if ‘[1,2]’ is selected:   ‘ConvLayer’ = ‘Denormalize(params, SC_Range, NB_Range, FS_Range, NF_Range)’
   ‘SC ∈ [1,2]’, ‘NB ∈ [2,4]’, ‘FS ∈ [2,8]’, ‘NF ∈ [4,128]’
5. Define ‘PoolLayer_Selection’:
 If ‘[1,1]’ is selected:
  ‘PoolLayer’ = ‘Denormalize(params, PC_Range, SP_Range, FS_Range, S_Range)’
   ‘PC ∈ [0,1]’, ‘SP ∈ [1,2]’, ‘FS ∈ [1,7]’, ‘S ∈ [1,3]’
6. Define ‘FCLayer_Selection’:
  ‘FCLayer’ = ‘Denormalize(params, SFC_Range, NN_Range)’
   ‘SFC ∈ [0,1]’, ‘NN ∈ [10,512]’
7. Generate and return ‘FinalOutput’

Step 2. Model Configuration and Hyperparameter Assignment: The values generated by the PSO algorithm are mapped to specific hyperparameter values through a denormalization process, ensuring a structured search within the predefined parameter space. The denormalization formula is as follows:

(8) $$V_{i}=round(L_{i}+\left(U_{i}-L_{i}\right)\times P_{i})$$

where $V_{i}$ denormalized value, $L_{i}$ and $U_{i}$ denote the lower and upper bounds of the parameter range, respectively, while $P_{i}$ represents the normalized parameter value within the range [0, 1], ensuring a structured mapping of the PSO-generated values to specific hyperparameter settings.

This transformation represents the first step in converting a continuous problem into a discrete one. While PSO operates in a continuous search space, this denormalization process ensures that values conform to discrete architectural constraints, allowing for the selection of distinct and unique CNN models. All hyperparameter ranges are defined in Step 1, allowing for flexible expansion or restriction of the search space based on specific task requirements. The PSO-normalized values are mapped to actual hyperparameter values using the predefined boundaries established in Step 1, ensuring that the architecture search remains scalable and adaptable. Figure 6c illustrates the auto-selective architecture design process, demonstrating how PSO-generated values undergo denormalization and are assigned to architectural components. The accompanying pseudocode further details this transformation, ensuring structured hyperparameter exploration and optimization.

With regard to its general structure (Figure 6), the SADASNet algorithm incorporates a variety of single-layer and double-layer operations for Selective CNN and Selective Class layers. Single-layer operations are performed in convolutional (Conv) layers, where denormalization is applied based on predefined constraints, including Selective Convolution (SC), Filter Size (FS), Number of Filters (NF), and Stride (S). Double-layer operations are executed in pooling (Pool) layers, where distinct denormalization methods are employed to optimize Pooling Control (PC), Selective Pooling type (SP), Filter Size (FS), and Stride (S). In the Selective Class layer, the Selective Fully Connected Control (SFC) and Number of Neurons (NN) are optimized through denormalization in fully connected (FC) layers. These denormalization processes are systematically applied within the parameter constraints specific to each layer type, ensuring an efficient and optimized execution of the algorithm. All these processes are outlined in detail in the pseudocode.

**Figure 7:**
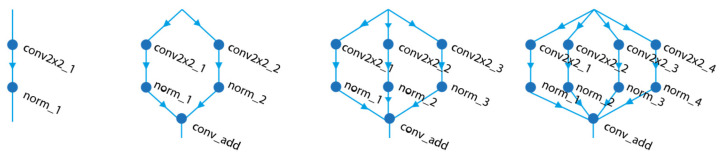
Multi branch structure for convulsion.

The selection of a convolutional layer is governed by the ConvLayer_Selection function. This structure incorporates a multi-branch configuration as an alternative to the standard convolution process, providing an opportunity for enhanced feature extraction. As illustrated in Figure 7, the multi-branch structure undergoes a sequence of processing steps, where selection options are derived from a predefined population, each corresponding to a specific convolutional operation. For instance, when the [1, 1] option is selected in Figure 6b, the standard convolution process is applied, with parameters such as Filter Size (FS), Number of Filters (NF), and Stride (S) being denormalized and utilized. In contrast, when the [1, 2] option is selected, the multi-branch structure is activated, incorporating additional parameters such as Number of Branches (NB), Filter Size (FS), and Number of Filters (NF) into the model. This flexible design allows for the integration of various convolutional layer configurations, thereby enhancing the feature extraction capabilities of the deep learning model.

The selection of the pooling layer is governed by the PoolLayer_Selection function, which determines whether a pooling operation should be included and specifies the type of pooling to be applied. As illustrated in Figure 6b, when the [5, 1] option is selected, standard pooling is enabled, and parameters such as Pooling Type (SP), Filter Size (FS), and Stride (S) are denormalized. This process defines the pooling operation (e.g., max pooling or average pooling) and assigns the appropriate hyperparameter values. Similarly, the selection of the fully connected (FC) layer is controlled by the FCLayer_Selection function. As shown in Figure 6b, selecting the [25, 1] option activates the fully connected layer, with the Number of Neurons (NN) parameter being denormalized to determine the layer’s configuration. These operations are systematically outlined in detail within the pseudocode.

In the model generation process, each model generated according to the selected population values is tested for the suitability of the layers for the hardware resources. This process involves the generation of different models in proportion to the population size, with the objective of optimizing the utilization of hardware resources. Each layer is subjected to a test before proceeding to the subsequent stage. In the event of an anomalous situation, the layer is removed from the model, thereby ensuring that the model functions correctly and without interruption.

Step 3. Calculation of Fitness Values: As illustrated in the provided graph, the initial dataset is divided into 80% for training and 20% for testing. While 90% of the training data are used for model training, the remaining 10% is set aside for validation. After these steps, the model undergoes both training and testing. During the testing phase, the model’s accuracy is measured, and the fitness function is calculated. The fitness function is used to define the loss value as follows:

(9) $$L=1-A_{model}$$

where L represents the loss value, reflecting the model’s prediction error, while $A_{model}$ denotes the model’s accuracy, indicating the proportion of correctly classified instances.

**Figure 8:**
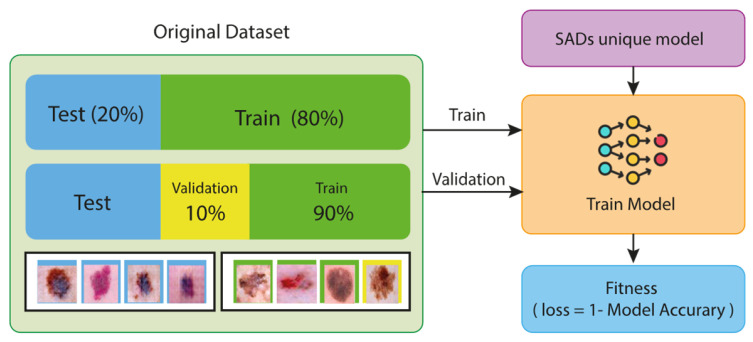
Training–testing phase of the proposed model.

The value of the fitness function is calculated using the generated model. This value represents the loss value of the model that is optimized within the scope of the SADASNet algorithm. Figure 8 illustrates the training and testing stages of the proposed model.

**Figure 9:**
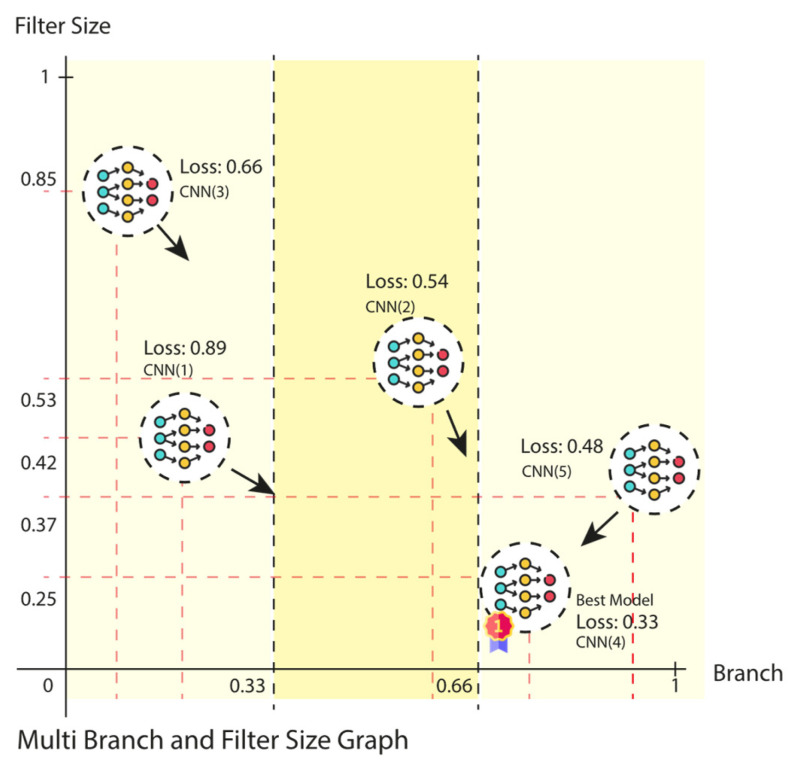
Movement of the PSO algorithm in layer selection.

Step 4. Particle Update: The SADASNet monitors the lowest loss value and the position of the model, and updates these values for each particle in accordance with the relevant parameters. In the event that the newly calculated loss value is less than that of the previous iteration, the current lowest loss value and the position of the particle are updated accordingly. The velocity of the particle is updated to move toward the target model. This update is designed to balance the competing objectives of ensuring that each particle moves toward its optimal position while simultaneously promoting the convergence of the entire population toward the best model. As illustrated in Figure 9, the CNN (4) model is identified as the best-performing model, and the filter size for the 4-branch convolution layer is determined to be 4. The remaining models then proceed to optimize their models by updating the number of branches and filters in the current layers toward the optimal position.

Step 5. Termination Check: These steps are repeated until a predetermined number of iterations has been reached or until a specific objective has been approached. In each iteration, the particles are updated in accordance with their lowest loss value and position.

Step 6. Final Evaluation: Upon completion of the SADASNet algorithm, the lowest loss value and the unique model are obtained. The specified CNN hyperparameter represents an approximation of the optimal solution to the optimization problem. Confusion Matrix, ROC-AUC, accuracy, precision, recall, F1-score, and Kappa results are obtained for the selected model evaluation.

#### Architectural Structures of the Models Obtained with SADASNet

**Figure 10:**
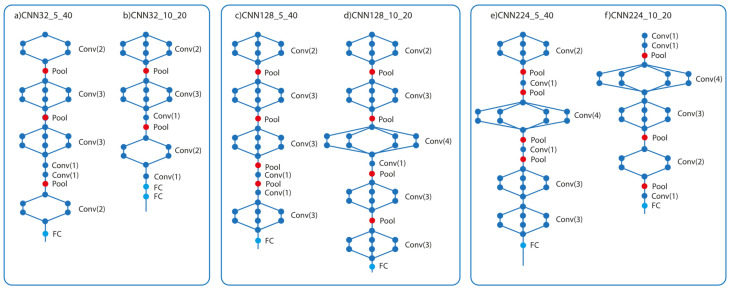
Architectures of the best models obtained with SADASNet.

In this study, experimental studies were conducted on HAM10000 dataset at three different resolutions: 32 × 32 × 3, 128 × 128 × 3, and 224 × 224 × 3. During the optimization phase, experiments were conducted with population sizes of 5 × 40 and 10 × 20. The names of the models obtained as a result of these experiments are presented in Table 1. The model names were initially designated according to the number of populations, number of iterations, image size, and model name. For instance, CNN32_10_20 denotes the designation of the optimal model obtained when the image size is 32 × 32 × 3, the number of populations is 10, and the iteration is 20. These models were subsequently evaluated on the augmented dataset, yielding a total of six distinct outcomes. Table 2 illustrates the layer parameters of the optimal CNN model generated with the SADASNet approach. The inclusion of this table is intended to provide comprehensive details regarding the model with the highest accuracy. Figure 10 presents the architectures of the best-performing models identified using the SADASNet framework, highlighting the network configurations that yielded optimal results.

## 4. Experimental Studies

This paper presents a novel approach to enhance the efficacy of automatic selective architecture design through the incorporation of hyperparameter optimization for the classification of skin lesions. The experimental studies were conducted using the MATLAB (MATLAB R2023b, MathWorks, Natick, MA, USA) platform. The MATLAB software was employed to implement deep learning and machine learning libraries in the experimental studies. The computer utilized for the experiments was equipped with an Intel(R) Core(TM) i5-8400 CPU @ 2.80 GHz (6 CPUs), 2.8 GHz, 16 GB RAM, and a GPU NVIDIA GeForce GTX 1080 with 8 GB memory.

The Stochastic Gradient Descent Momentum (SGDM) optimization algorithm and a graphical processing unit (GPU) were employed to train the model. The initial learning rate was set to 0.0001, and the maximum number of training epochs was set to 50. The mini-batch size is contingent upon the hardware and is defined as the default value of 32. The ‘piecewise’ method is the preferred learning rate timing. The learning rate was reduced by 20% every 5 epochs. A value of 0.0001 was used for L2 regularization. The validation dataset (validation and validation Label) and validation frequency were set to every 50 iterations.

### 4.1. Performance of SADASNet on ISIC Dataset of Different Image Sizes

This comprehensive study systematically evaluates the performance of SADASNet on the HAM10000 dataset at 32 × 32, 128 × 128, and 224 × 224 resolutions, demonstrating the critical interplay between scalability, hardware compatibility, and model stability in deep learning-based skin cancer classification. The experimental methodology is designed to ensure fair cross-resolution comparisons, optimize GPU memory utilization (5-epoch training time < 30 min, GPU memory consumption < 8 GB), and establish a scalable and consistent framework for model selection. To meet these criteria, a strategically selected subset of 200 instances per class was employed. Experimental evaluations demonstrated that this sample size achieves an optimal balance between computational efficiency, statistical representativeness, and training stability. This selection enhanced the traceability of early-stage training, accelerated model discovery, and ensured stable and optimized performance across different resolutions. Accordingly, a two-stage training strategy was implemented: in the first stage, candidate architectures were rapidly evaluated over 5 epochs using 200 images, followed by an extended 50-epoch training on the full dataset for the highest-performing model (CNN224_5_40: 4.4 M parameters, 3.77 B FLOPs). SADASNet demonstrated robust and consistent performance, achieving 98.53–99.31% accuracy and 95.11–97.58% F1-score across different resolutions, while selecting hardware-optimized architectures within 2.2 M–9.6 M parameters and 0.44 B–11.52 B FLOPs, ensuring an optimal balance between computational efficiency and predictive accuracy. Throughout this process, model selection was optimized with respect to hardware compatibility and computational efficiency. This approach establishes an experimental framework for designing scalable and robust systems even under resource constraints. Detailed performance analyses are presented in Section 4.1.1, Section 4.1.2 and Section 4.1.3.

#### 4.1.1. Performance Evaluation on 32 × 32 Image Size

**Figure 11:**
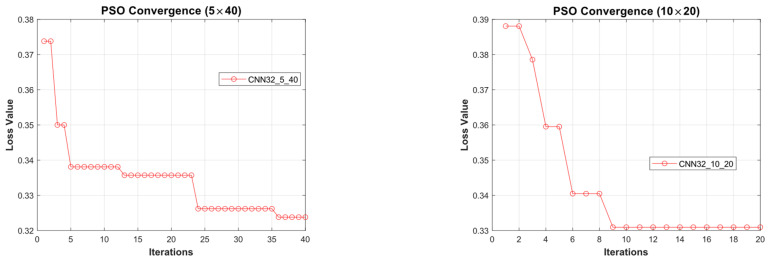
Convergence graph of PSO algorithm for CNN32_5_40 and CNN32_10_20.

**Figure 12:**
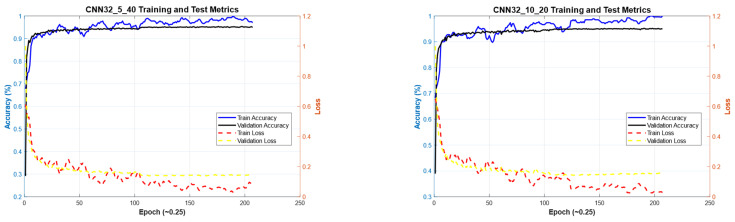
Convergence graphs of CNN32_5_40 and CNN32_10_20 models on the dataset during the training phase.

**Figure 13:**
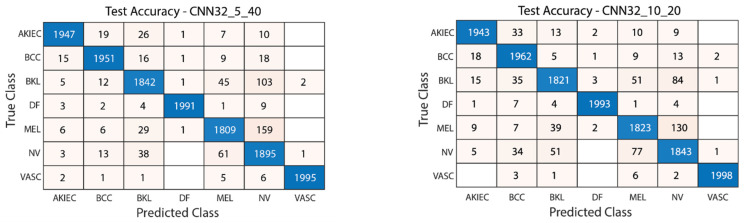
Confusion matrix of CNN32_5_40 and CNN32_10_20.

**Figure 14:**
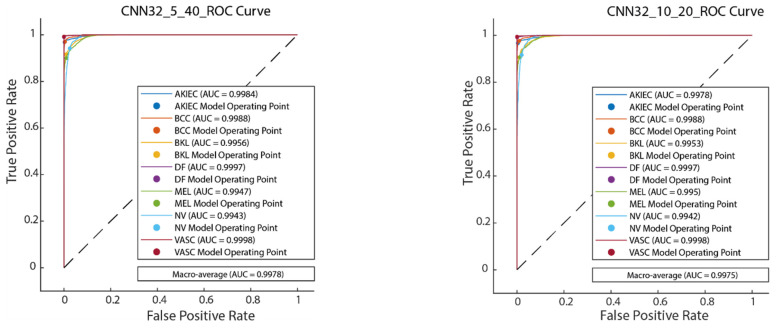
ROC-AUC plot of CNN32_5_40 and CNN32_10_20.

Figure 11 illustrates the convergence graph of the PSO algorithm utilized for the optimization process. Furthermore, the convergence graph of the model obtained in the CNN32_5_40 and CNN32_10_20 training phase is provided in Figure 12. The confusion matrix is illustrated in Figure 13, and the ROC-AUC graph is depicted in Figure 14. The performance metrics obtained by the model on the HAM10000 dataset are presented in Table 3.

#### 4.1.2. Performance Evaluation on 128 × 128 Image Size

**Figure 15:**
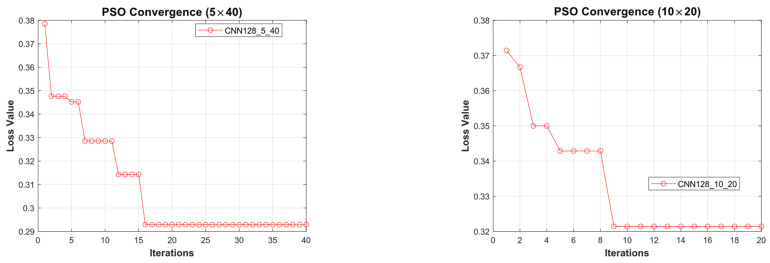
Convergence graph of PSO algorithm for CNN128_5_40 and CNN128_10_20.

**Figure 16:**
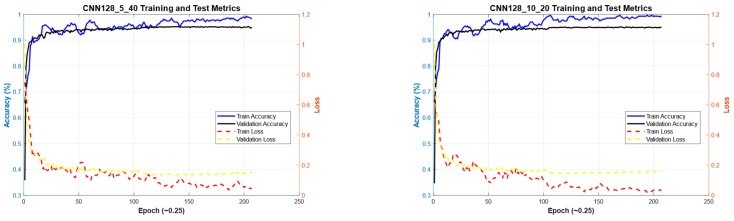
Convergence graphs of CNN128_5_40 and CNN128_10_20 models on the dataset during the training phase.

**Figure 17:**
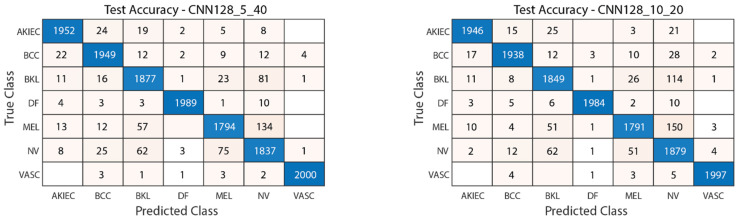
Confusion matrix of CNN128_5_40 and CNN128_10_20.

**Figure 18:**
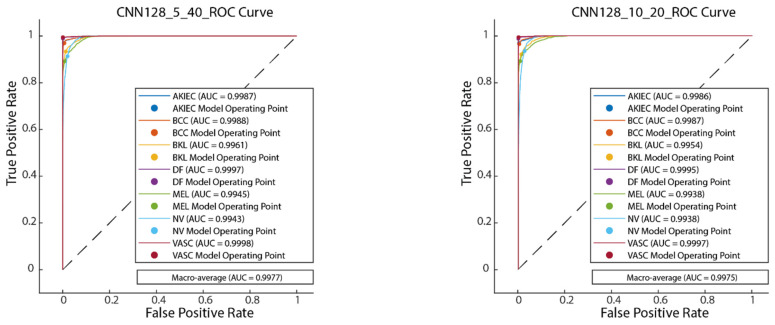
ROC-AUC plot of CNN128_5_40 and CNN128_10_20.

Figure 15 illustrates the convergence graph of the PSO algorithm utilized for the optimization process. Furthermore, the convergence graph of the model obtained in the CNN128_5_40 and CNN128_10_20 training phase is provided in Figure 16. The confusion matrix is illustrated in Figure 17, and the ROC-AUC graph is depicted in Figure 18. The performance metrics obtained by the model on the HAM10000 dataset are presented in Table 4.

#### 4.1.3. Performance Evaluation on 224 × 224 Image Size

**Figure 19:**
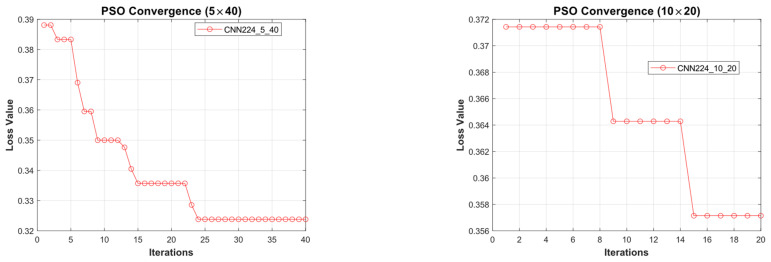
Convergence graph of PSO algorithm for CNN224_5_40 and CNN224_10_20.

**Figure 20:**
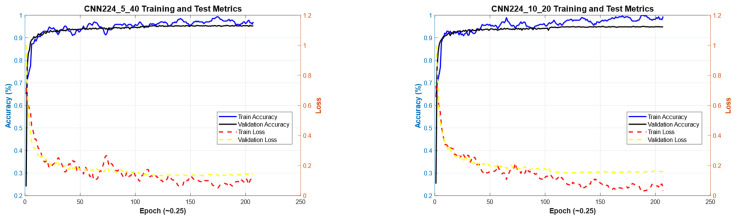
Convergence graphs of CNN224_5_40 and CNN224_10_20 models on the dataset during the training phase.

**Figure 21:**
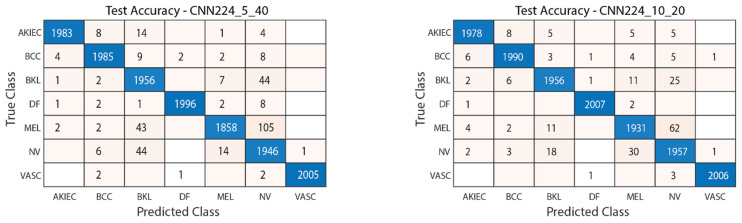
Confusion matrix of CNN224_5_40 and CNN224_10_20.

**Figure 22:**
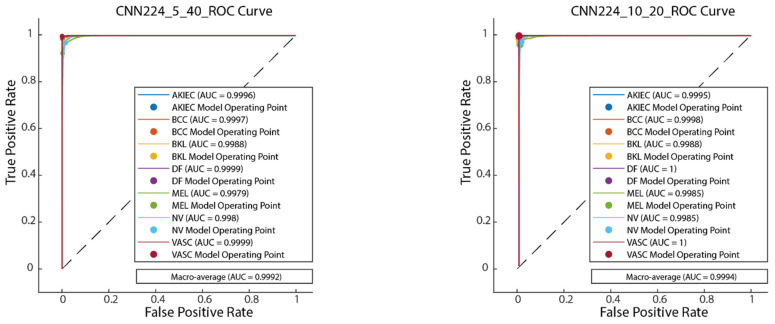
ROC-AUC plot of CNN224_5_40 and CNN224_10_20.

Figure 19 illustrates the convergence graph of the PSO algorithm utilized for the optimization process. Furthermore, the convergence graph of the model obtained in the CNN224_5_40 and CNN224_10_20 training phase is provided in Figure 20. The confusion matrix is illustrated in Figure 21, and the ROC-AUC graph is depicted in Figure 22. The performance metrics obtained by the model on the HAM10000 dataset are presented in Table 5.

#### 4.1.4. In Summary: Performance Evaluation Across Three Different Image Resolutions

**Figure 23:**
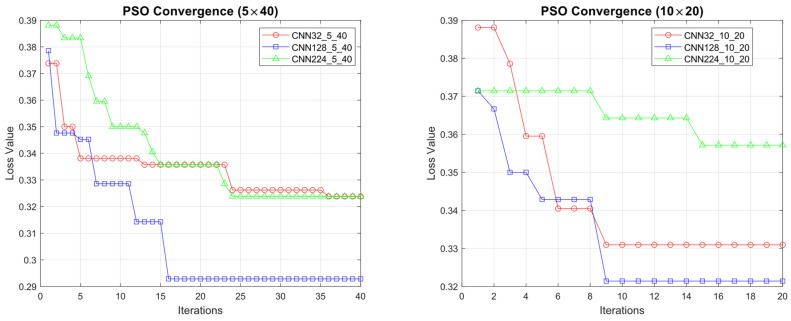
Convergence curves of the PSO algorithm for different CNN configurations (5 × 40 vs. 10 × 20).

**Figure 24:**
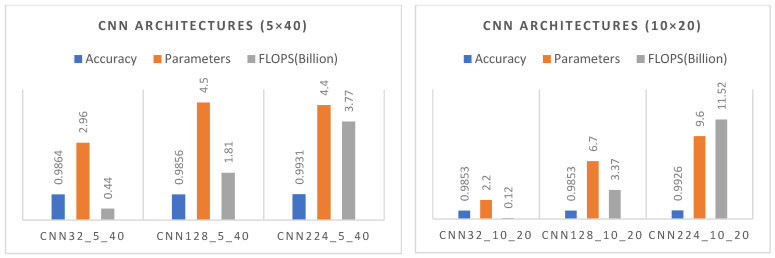
Comparison of accuracy, number of parameters, and FLOPs in CNN models with 5 × 40 and 10 × 20 layer configurations.

As shown in Figure 23, the convergence curves of the PSO algorithm exhibit a similar pattern for both the 5 × 40 and 10 × 20 CNN configurations, with a slightly faster initial descent in the 5 × 40 structure. Meanwhile, Figure 24 offers a comprehensive comparison of accuracy, parameter count, and FLOPs, thereby illustrating the inherent balance between model performance and computational overhead.

In this work, we evaluate the performance metrics of various CNN architectures trained at three resolution levels, 32 × 32, 128 × 128, and 224 × 224, to achieve an optimal balance between computational efficiency and accuracy. The findings show that at a resolution of 32 × 32, faster and more compact models provide satisfactory performance with minimal computational overhead. At 128 × 128, accuracy is maintained by incorporating additional visual details. Finally, at 224 × 224, the models reach their highest performance in terms of accuracy and generalization; in particular, CNN224_5_40 achieves 99.31% accuracy and an F1 score of 0.9758, while maintaining a relatively efficient structure in terms of parameters and FLOPs. This analysis underscores the importance of selecting a CNN architecture suited for the target application domain and hardware constraints, offering valuable guidance for deep learning–based classification tasks.

### 4.2. Performance Evaluation of the Models Obtained by SADASNet

The results demonstrate the rapid convergence capability of the PSO algorithm and indicate that the 5 × 40 configuration exhibits lower error rates and a more stable convergence process than the 10 × 20 configuration (Figure 11, Figure 14 and Figure 19). In this process, it is observed that in both models, the PSO algorithm rapidly reduces the error rates to a significant extent after approximately 10 iterations for the 5 × 40 configuration. To illustrate, the CNN32_5_40 model reduced the error rate from 0.38 to 0.32, while the CNN128_5_40 model decreased from 0.38 to 0.31, and the CNN224_5_40 model reduced the error rate from 0.39 to 0.325. These findings substantiate the efficacy of the PSO algorithm as a means of optimizing hyperparameters in DL models, thereby enhancing model performance. Moreover, the CNN32_5_40, CNN128_5_40, and CNN224_5_40 models, obtained with the 5 × 40 configuration, demonstrated superior accuracy and lower loss function values on both training and test data (Figure 12, Figure 16 and Figure 20). A comprehensive analysis was conducted using complexity matrices (Figure 13, Figure 17 and Figure 21), ROC curves (Figure 14, Figure 18 and Figure 22), and class-based performance metrics (Table 4, Table 5 and Table 6). The findings revealed that the 5 × 40 model demonstrated superior classification performance compared to the 10 × 20 model. Upon analysis of the ROC curves and AUC values, it becomes evident that both models exhibit high AUC values (typically above 0.98), signifying their effectiveness in discerning between the true positive and negative classes. These findings indicate that the 5 × 40 configuration, which performs a more comprehensive and exhaustive search, is more effective in determining optimal hyperparameter values, resulting in superior model performance. The results demonstrate that the PSO algorithm is an effective approach for hyperparameter optimization of deep learning models, with more extensive searches leading to more successful outcomes. Consequently, this study highlights the importance of carefully selecting and optimizing the configuration of the PSO algorithm to maximize the performance of deep learning models, taking into account the complexity of the dataset and model.

The parameter, layer, and FLOPs values of the proposed method and the success metrics obtained on the dataset are shown together in Table 6.

Table 6 assesses the influence of disparate image dimensions and model configurations on the performance metrics of the models developed with the GAN dataset. The results demonstrate that models operating with larger image sizes (e.g., CNN224_5_40 and CNN224_10_20) demonstrate superior performance, exhibiting high accuracy (99.31%) and specificity (99.26%). These findings indicate that large image sizes and complex structures are crucial for enhancing classification performance. Nevertheless, it is noteworthy that smaller models (based on CNN32 and CNN128) can also yield satisfactory results and may serve as a viable option when minimizing parameter and computational cost is a priority. In particular, the CNN32_10_20 model exhibits high efficiency with a minimal number of parameters (2.2 M), underscoring the significance and adaptability of architectural exploration. This demonstrates that the SADASNet algorithm is capable of not only achieving high accuracy but also of producing optimized models that can adapt to different computational requirements. Accordingly, the selection of appropriate sizes and structures, in accordance with the intended application and available resources, is of paramount importance in the architectural exploration process. Furthermore, this study optimized and analyzed the FLOPs and parameter numbers of models trained with 32 × 32 × 3, 128 × 128 × 3, and 224 × 224 × 3 input images. The results demonstrate that distinct optimizations for an identical image size have a direct impact on the parameter size and computational cost of the model. Consequently, it is imperative to adopt a balanced approach between model performance and computational cost when optimizing hyperparameters.

### 4.3. Comparative Analysis

**Figure 25:**
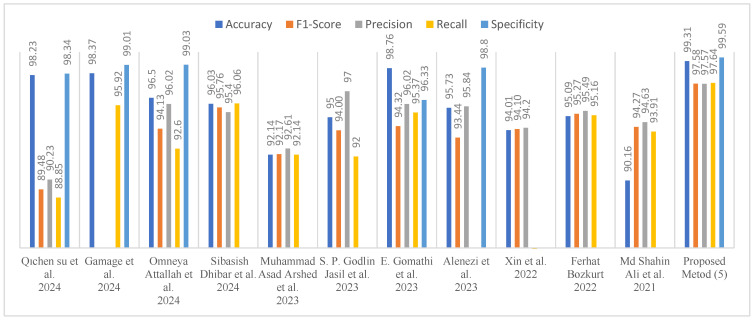
Comparison analysis [12,14,15,16,17,30,31,32,33,34,35].

Several studies have been conducted using the HAM10000 dataset. The most optimal model results from these studies are compared with the outcomes achieved through the proposed methodology, as shown in Table 7. Figure 25 offers a comprehensive comparative analysis of the proposed method in relation to the performance metrics of various studies in the existing literature.

Table 7 presents a comparative analysis of the proposed method with existing works for the classification of skin cancer images. The objective of this table is to facilitate a comparative analysis of the performance of the proposed method with that of other existing methods. The table includes a number of performance measures, including accuracy, F1-score, precision, recall, and specificity, which are commonly employed in the relevant literature. The first column enumerates prior studies and the methodologies employed therein. The second column provides a detailed description of each methodology, including the models developed by the respective authors. The third column specifies the dataset used in each study, while the fourth and fifth columns indicate whether segmentation and data augmentation techniques were applied. As the deployment of these techniques can markedly influence the outcomes, the disclosure of this information is crucial for performance comparisons. As illustrated in Table 7, the performance of the proposed SADASNet algorithm is superior to that of numerous state-of-the-art models. Notably, SADASNet Method 5 achieved an accuracy of 99.31% utilizing the CNN224_5_40 architecture in combination with a GAN-based data augmentation technique. This result is comparable to those of other high-performance methods, including STGAN (98.23%), as proposed by [12], and DODL (98.76%), as proposed by [17]. The SADASNet algorithm reduces the complexity of the model by optimizing critical hyperparameters, including the number of deep network layers, convolution parameters, pooling parameters, fully connected layer parameters, activation functions, and dropout rates. This approach allows for the achievement of high accuracy rates using fewer parameters, thereby improving computational efficiency. The SADASNet algorithm reduces the complexity of the model and increases computational efficiency, thereby rendering it applicable even in the presence of limited hardware resources. This method offers significant advantages in practical applications by eliminating the necessity for the use of overly complex models with a large number of parameters in order to achieve high accuracy rates. The model’s segmentation-free structure allows for a more efficient solution with a reduced computational cost. Moreover, the use of GAN-based data augmentation enhances the model’s flexibility and reduces the error rate. These findings underscore the significance of prioritizing architectural exploration over intricate segmentation or data augmentation. The proposed method demonstrates that CNN architectures devised with distinctive serial and parallel layer configurations can effectively encapsulate intricate skin lesion characteristics, thereby enabling the attainment of cutting-edge outcomes for precise diagnosis and classification of skin lesions.

## 5. Discussion

In recent years, DL models have demonstrated significant efficacy in the classification of skin cancers. However, the large number of hyperparameters inherent to CNN models, whether manually designed or trained via transfer learning, poses a substantial challenge in achieving optimal performance. In this study, the PSO-based Selective and Adaptive Deep Architecture Search Network by Hyperparameter Optimization (SADASNet) algorithm is employed to develop high-performance CNN models with a distinctive serial and parallel structure. The objective of the SADASNet algorithm is to achieve superior results in a reduced number of iterations while minimizing the computational cost by leveraging the rapid convergence characteristic of PSO. The SADASNet algorithm generates more sophisticated and optimized models by concurrently optimizing both parallel and serial layer structures and the hyperparameters of CNNs.

Moreover, the SADASNet algorithm, leveraging PSO-based optimization and an independent training principle, eliminates common issues seen in other methods, such as indirect performance evaluation challenges, by directly assessing the true performance of each candidate architecture. This approach ensures higher accuracy and reliability while offering an effective and alternative perspective in NAS processes.

Consequently, the SADASNet algorithm exhibits high accuracy rates in skin cancer classification and provides more suitable and reliable solutions for clinical applications. The comprehensive analysis presented in Table 7 provides an in-depth evaluation of the performance and technical characteristics of existing work and the proposed SADASNet algorithm. This comparison is based on data augmentation techniques, segmentation requirements, complexity of architectural structures, and performance metrics used in the field of skin cancer diagnosis and classification.

Numerous studies utilize various data augmentation techniques to address data imbalance and improve classification performance. In a recent study, [12] proposed a novel approach, Self-Transfer GAN (STGAN), which employs a GAN-based methodology to address the issue of data imbalance. Reference [14] sought to enhance classification efficacy by leveraging conventional image transformations within the Mask-Guided ViT-GradCAM model. Reference [17] incorporated segmentation procedures into the data augmentation process with the DODL model, whereas studies such as [31,32] conducted data augmentation with image transformations. In contrast to other approaches, the SADASNet algorithm does not necessitate additional preprocessing or segmentation steps, as it employs a GAN-based data augmentation technique to address data imbalance. This distinguishes the SADASNet algorithm as a more straightforward and computationally efficient solution.

Methods that necessitate segmentation frequently result in augmented computational costs and complexity. For example, the DODL model developed by [17] employs U-Net-based segmentation, whereas the Skin-CAD model developed by [15] attains high accuracy rates by integrating segmentation techniques with intricate CNN architectures. In contrast, the SADASNet algorithm, which lacks a segmentation structure, is free from these additional complexities and provides a more efficient solution. This is a significant advantage, particularly in resource-constrained clinical applications.

Some studies make use of intricate architectural designs that necessitate a considerable expenditure of computational resources. Refs. [16,33] employed InceptionResNetV2 and wavelet transform-based networks with substantial parameter sets and intricate structures. Conversely, Refs. [34,35] devised computationally demanding transformer-based networks. In contrast, the SADASNet algorithm provides flexibility to explore architectural options, optimizing models with varying parameter counts, and accommodating diverse data sizes (32, 128, and 224). This adaptability enables the SADASNet algorithm to accommodate a diverse range of applications and to operate effectively with a spectrum of computational resources.

Performance evaluations are based on a set of metrics, including accuracy (%), F1-Score, recall, precision, and specificity. Reference [12] achieved an accuracy of 98.23%, Ref. [14] 98.37%, Ref. [15] 96.50%, and Reference [32] were 97% accurate, while [33] achieved 95.09% accuracy. Additionally, Ref. [16] attained 96.91% accuracy. The proposed SADASNet algorithm exhibited the highest level of performance, achieving an accuracy of 99.31%, an F1 score of 97.58%, a recall of 97.57%, and a precision of 97.64%. Notably, with a specificity of 99.59%, the SADASNet algorithm effectively minimized false positive rates, offering a reliable solution for clinical applications. These metrics indicate that the SADASNet algorithm provides a more balanced and consistent performance compared to other methods.

The SADASNet represents an innovative and adaptable solution for automated CNN architecture search, delivering high accuracy and computational efficiency in skin cancer classification. By integrating PSO-based hyperparameter optimization with GAN-supported data augmentation, it effectively mitigates data imbalance while seamlessly adapting to varying image resolutions and hardware constraints. Unlike traditional methods that employ fixed layer configurations, SADASNet automatically generates both serial and multi-branch parallel layer structures, thereby broadening the scope of model discovery and enabling the identification of the most suitable architecture for a given problem. Its segmentation-free, low-parameter design not only preserves diagnostic accuracy but also enhances applicability in resource-constrained environments, making advanced deep learning techniques accessible to users with limited expertise.

Furthermore, SADASNet is a dataset-independent architecture that autonomously discovers and dynamically optimizes the most effective CNN model tailored to the characteristics of any dataset. In datasets where skin tones are well balanced, it discovers the optimal architecture to achieve high accuracy. Unlike traditional models with fixed architectures, SADASNet dynamically optimizes its structure based on the available data, avoiding performance degradation and continuously refining architectures to discover the most effective design for each unique dataset.

Despite its promising performance, the proposed approach has several limitations. Variations in image quality, resolution, noise, and lighting inconsistencies can reduce lesion visibility, impacting classification accuracy. The exclusive use of the RGB color space limits feature extraction, preventing the model from leveraging alternative color representations like HSV or LAB. High computational demands require substantial GPU memory, increasing training time and limiting scalability, especially in resource-constrained environments. Also, the proposed method has been trained on the publicly available HAM10000 dataset and has not yet been validated in real-world clinical settings. As a result, its practical implementation and ethical implications in clinical practice require further investigation.

Future research will focus on expanding dataset diversity, integrating advanced color spaces, improving optimization strategies, and adopting adaptive learning techniques to enhance model performance.

## 6. Conclusions

This paper introduces the Selective and Adaptive Deep Architecture Search Network by Hyperparameter Optimization (SADASNet) algorithm, an innovative approach to skin cancer classification, and presents an extensive evaluation of its performance. Despite the growing use of artificial intelligence and DL in skin cancer classification, the majority of studies continue to rely on hand-designed CNN models or transfer learning techniques, which often struggle to achieve high accuracy due to the numerous parameters that require optimization. To address these limitations, we have developed the SADASNet algorithm, which integrates a PSO-based metaheuristic optimization method. The SADASNet algorithm employs the rapid convergence potential of PSO to concurrently optimize both serial and parallel CNN layers. This process automates steps such as configuring layers and tuning hyperparameters, thereby facilitating a more efficient architecture exploration process. GAN-based data augmentation techniques are employed to expand the dataset, thereby enabling the model to perform optimally with limited data and to achieve balanced results with more diverse data. This integrated approach enabled the SADASNet algorithm to achieve high accuracy rates in fewer iterations while reducing computational costs. The performance of the SADASNet algorithm was evaluated on the HAM10000 dataset, achieving high metrics, including 99.31% accuracy, 97.58% F1 score, 97.57% recall, and 97.64% precision. Notably, the specificity value of 99.59% demonstrates the algorithm’s reliability in clinical applications by minimizing false positive rates. In contrast to alternative methodologies, the SADASNet algorithm offers efficacious solutions to data imbalance issues without the necessity for segmentation or supplementary preprocessing procedures. This evidence substantiates the flexible and feasible nature of the SADASNet algorithm and indicates its potential for extensive utilization in clinical applications. In conclusion, the SADASNet algorithm offers an optimized and rapid solution for skin cancer diagnosis, combining high accuracy rates with low computational costs. This study demonstrates how the strategic design of deep learning architecture plays a critical role in improving accuracy and efficiency in medical diagnostic systems. These advantages of the SADASNet algorithm make it possible to achieve successful results in different application areas and even in resource-constrained situations.

## Figures and Tables

**Table 1:** Number of populations, number of iterations, image size, and model nomenclature of the models proposed in studies with SADASNet with different parameters.

Population Number	Number of Iteration	Image Size	Model Name
5	40	32 × 32	CNN32_5_40
10	20	32 × 32	CNN32_10_20
5	40	128 × 128	CNN128_5_40
10	20	128 × 128	CNN128_10_20
5	40	224 × 224	CNN224_5_40
10	20	224 × 224	CNN224_10_20

**Table 2:** Parameters in each layer of the CNN224_5_40 model.

No.	Layer	Activations	Number of Filter	Filter Size	Stride	Total Parameter
1	‘CNN_conv2 × 2_1_Conv_1’ 2-D Convolution	224 × 224	87	1 × 1	1 × 1	348
2	‘CNN_conv2 × 2_1_Conv_2’ 2-D Convolution	224 × 224	87	3 × 3	1 × 1	2436
3	‘CNN_conv2 × 2_1_Conv_3’ 2-D Convolution	224 × 224	87	5 × 5	1 × 1	6612
4	‘CNN_pool_2’ 2-D Max Pooling	110 × 110	261	1 × 1	2 × 2	0
5	‘CNN_conv_3’ 2-D Convolution	55 × 55	112	6 × 6	2 × 2	1,052,464
6	‘CNN_pool_4’ 2-D Max Pooling	26 × 26	112	4 × 4	2 × 2	0
7	‘CNN_conv2 × 2_5_Conv_1’ 2-D Convolution	26 × 26	102	1 × 1	1 × 1	11,526
8	‘CNN_conv2 × 2_5_Conv_2’ 2-D Convolution	26 × 26	102	3 × 3	1 × 1	102,918
9	‘CNN_conv2 × 2_5_Conv_3’ 2-D Convolution	26 × 26	102	5 × 5	1 × 1	285,702
10	‘CNN_conv2 × 2_5_Conv_4’ 2-D Convolution	26 × 26	102	7 × 7	1 × 1	559,878
11	‘CNN_pool_6’ 2-D Max Pooling	12 × 12	408	4 × 4	2 × 2	0
12	‘CNN_conv_7’ 2-D Convolution	6 × 6	144	6 × 6	2 × 2	2,115,216
13	‘CNN_pool_8’ 2-D Max Pooling	2 × 2	144	4 × 4	2 × 2	0
14	‘CNN_conv2 × 2_9_Conv_1’ 2-D Convolution	2 × 2	41	1 × 1	1 × 1	5945
15	‘CNN_conv2 × 2_9_Conv_2’ 2-D Convolution	2 × 2	41	3 × 3	1 × 1	53,177
16	‘CNN_conv2 × 2_9_Conv_3’ 2-D Convolution	2 × 2	41	5 × 5	1 × 1	147,641
17	‘CNN_conv2 × 2_11_Conv_1’ 2-D Convolution	2 × 2	28	1 × 1	1 × 1	3472
18	‘CNN_conv2 × 2_11_Conv_2’ 2-D Convolution	2 × 2	28	3 × 3	1 × 1	31,024
19	‘CNN_conv2 × 2_11_Conv_3’ 2-D Convolution	2 × 2	28	5 × 5	1 × 1	86,128
20	‘FCLayer’	1 × 1	7	1 × 1	1 × 1	595
						4.4 M

**Table 3:** Performance metrics of CNN32_5_40 and CNN32_10_20 for each class.

	CNN32_5_40 (2.96 M Parameter)				CNN32_10_20 (6.4 M Parameter)			
	Precision	Recall	F1-Score	Accuracy	Precision	Recall	F1-Score	Accuracy
AKIEC	0.9828	0.9686	0.9757	0.9927	0.9793	0.9627	0.9709	0.9913
BCC	0.9735	0.9706	0.9721	0.9916	0.9677	0.9682	0.9679	0.9903
BKL	0.9417	0.9164	0.9289	0.9790	0.9227	0.9139	0.9183	0.9755
DF	0.9979	0.9905	0.9942	0.9982	0.9945	0.9905	0.9925	0.9978
NV	0.9339	0.9000	0.9166	0.9755	0.9311	0.8940	0.9122	0.9741
VASC	0.8613	0.9423	0.9000	0.9686	0.8612	0.9259	0.8924	0.9664
MEL	0.9985	0.9925	0.9955	0.9986	0.9970	0.9915	0.9943	0.9983

**Table 4:** Performance metrics of CNN128_5_40 and CNN128_10_20 for each class.

	CNN128_5_40 (4.5 M Parameter)				CNN128_10_20 (6.7 M Parameter)			
	Precision	Recall	F1-Score	Accuracy	Precision	Recall	F1-Score	Accuracy
AKIEC	0.9711	0.9711	0.9711	0.9913	0.9788	0.9686	0.9737	0.9921
BCC	0.9591	0.9696	0.9643	0.9892	0.9658	0.9696	0.9677	0.9903
BKL	0.9241	0.9338	0.9289	0.9785	0.9229	0.9293	0.9261	0.9777
DF	0.9955	0.9895	0.9925	0.9977	0.9964	0.9865	0.9915	0.9974
NV	0.9392	0.8925	0.9153	0.9752	0.9507	0.8930	0.9209	0.9770
VASC	0.8814	0.9134	0.8971	0.9685	0.8632	0.9264	0.8937	0.9669
MEL	0.9970	0.9950	0.9960	0.9988	0.9975	0.9945	0.9960	0.9988

**Table 5:** Performance metrics of CNN224_5_40 and CNN224_10_20 for each class.

	CNN224_5_40 (4.4 M Parameter)				CNN224_10_20 (9.6 M Parameter)			
	Precision	Recall	F1-Score	Accuracy	Precision	Recall	F1-Score	Accuracy
AKIEC	0.99598	0.98657	0.99125	0.99751	0.9925	0.9885	0.9905	0.9973
BCC	0.98904	0.98756	0.9883	0.99666	0.9805	0.9800	0.9803	0.9922
BKL	0.9463	0.97313	0.95953	0.98827	0.9515	0.9730	0.9565	0.9841
DF	0.9985	0.99303	0.99576	0.99879	0.9985	0.9935	0.9965	0.9995
NV	0.9862	0.92438	0.95429	0.98735	0.9737	0.9340	0.9571	0.9906
VASC	0.91923	0.96768	0.94283	0.98323	0.9313	0.9621	0.9501	0.9850
MEL	0.9995	0.99751	0.99851	0.99957	0.9990	0.9980	0.9985	0.9995

**Table 6:** Performance metrics obtained for the original and generated dataset.

Proposed Method	Model Name	Accuracy	F1-Score	Recall	Precision	Specificity	Number of Parameters (M)	Layers	FLOPs(B)
1	CNN32_5_40	0.9864	0.9547	0.9544	0.9557	0.9920	2.96	48	0.44
2	CNN32_10_20	0.9853	0.9511	0.9511	0.9515	0.9914	2.2	60	0.12
3	CNN128_5_40	0.9856	0.9522	0.9522	0.9525	0.9916	4.5	52	1.81
4	CNN128_10_20	0.9853	0.9515	0.9512	0.9527	0.9916	6.7	60	3.37
5	**CNN224_5_40**	**0.9931**	**0.9758**	**0.9757**	**0.9764**	**0.9959**	**4.4**	**54**	**3.77**
6	CNN224_10_20	0.9926	0.9757	0.9756	0.9753	0.9953	9.6	45	11.52

**Table 7:** Performance metrics of the methods applied on the HAM10000 dataset (study (year), method, dataset, segmentation, data augmentation and performance metrics).

Study (Year)	Method	DataSet	Segmentation	Data Augmentation	Performance Metrics
Qichen su et al. [12]	A GAN-based data augmentation Self-transfer GAN (STGAN)	HAM 10000 (7 class)	NO	GAN	Accuracy:98.23% F1-Score:89.48% Recall:88.85% Precision:90.23% Specificity:98.34%
Gamage et al. [14]	Mask-Guided ViT-GradCAM for Skin Lesion Classification	HAM 10000 (7 class)	YES	Image Transformations	Accuracy: 98.37% Specificity: 99.01% Recall: 95.92%
Omneya Attallah et al. [15]	Skin-CAD: Explainable deep learning classification (Inception + Xception + ResNet-50 + ResNet-101 + Relief-F)	HAM 10000 (7 class)	YES	Image Transformations	Accuracy: 96.50% Recall: 92.60% F1-score: 94.13% Precision: 96.02% Specificity: 99.03%
Sibasish Dhibar et al. [30]	DAE-ResNet101-based SC classification model	HAM 10000 (7 class)	NO	Image Transformations	Accuracy: 96.03% F1 score: 95.76% Recall: 96.06% Precision: 95.40%
Muhammad Asad Arshed et al. [31]	Vision transformers (RGB Images)	HAM 10000 (7 class)	NO	Image Transformations	Accuracy: 92.14% F1 score: 92.17% Recall: 92.14% Precision: 92.61%
S. P. Godlin Jasil et al. [32]	A hybrid CNN architecture (Densenet and residual network)	HAM 10000 (7 class)	NO	Image Transformations	Accuracy: 95% F1 score: 94% Recall: 92% Precision: 97%
E. Gomathi et al. [17]	DODL (dual optimization) with U-net segmentation	HAM 10000 (7 class)	YES	Image Transformations	Accuracy: 98.76% F1 score: 94.32% Recall: 95.37% Precision: 96.02% Specificity: 96.33%
Ferhat Bozkurt [33]	Augmentation + InceptionResNetV2	HAM 10000 (7 class)	NO	Image Transformations	Accuracy: 95.09% F1 score: 95.27% Recall: 95.16% Precision: 95.49%
Alenezi et al. [16]	Wavelet transform-based Deep Residual Neural Network (WT-DRNNet)	HAM 10000 (7 class)	NO	Image Transformations	Accuracy: 95.73% F1 score: 93.44% Precision: 95.84% Specificity: 98.80%
Xin et al. [34]	Improved transformer network	HAM 10000 (7 class)	NO	Image Transformations	Accuracy: 94.1% F1 score: 94.1% Precision: 94.2%
Md Shahin Ali et al. [35]	Deep Convolutional Neural Networks (DCNNs)	HAM 10000 (7 class)	NO	Image Transformations	Accuracy: 90.16% F1 score: 94.27% Recall: 93.91% Precision: 94.63%
**Proposed Method 5** **2024**	**Selective and Adaptive Architectural Search (SADASNET)** **CNN224_5_40**	**HAM 10000** **(7 class)**	**NO**	**GAN**	**Accuracy: 99.31%** **F1-Score: 97.58%** **Recall: 97.57%** **Precision: 97.64%** **Specificity: 99.59%**

## Data Availability

The dataset was obtained from [13].
